# Do personalised e-mail invitations increase the response rates of breast cancer survivors invited to participate in a web-based behaviour change intervention? A quasi-randomised 2-arm controlled trial

**DOI:** 10.1186/s12874-015-0063-5

**Published:** 2015-08-19

**Authors:** Camille E. Short, Amanda L. Rebar, Corneel Vandelanotte

**Affiliations:** Freemasons Foundation Centre of Men’s Health, School of Medicine, University of Adelaide, Level 7, South Australian Health and Medical Research Institute, North Terrace, Adelaide, 5000 Australia; Physical Activity Research Group, School of Human Health and Social Sciences, Central Queensland University, Building 18, Bruce Highway, Rockhampton, QLD 4702 Australia

## Abstract

**Background:**

Previous research has shown that the personalisation of study invitations improves response rates in survey-based research. To examine if this finding extends to experimental studies, we examined the impact of personalised study invitation e-mails on the response rates of potentially eligible breast cancer survivors for participation in a 6 month randomised controlled trial testing the efficacy of a physical activity intervention.

**Methods:**

Potential participants (*n* = 344) were sent either a personalised email or a generic email.

**Results:**

Those sent the personalised email were 1.5 times (95 % CI = 1.18–1.93) more likely to respond than those sent the generic email.

**Conclusion:**

These findings suggest that personalisation may be a useful and potentially powerful tool that can be utilised when recruiting participants into experimental studies in order to boost response rates.

**Electronic supplementary material:**

The online version of this article (doi:10.1186/s12874-015-0063-5) contains supplementary material, which is available to authorized users.

## Background

The potential of online behaviour change interventions for improving public health in both a primary and tertiary prevention setting is well recognised [1, 2]. Due to this, and the growing popularity of the internet, the last decade has seen a substantial increase in the number of online behaviour change interventions developed and evaluated [3, 4]. Several well-conducted systematic reviews and meta-analyses have synthesised the literature regarding this research [1, 3, 5–9]. In general, these reviews have shown that online interventions can be effective (albeit effect sizes have been small), but that issues with recruiting and retaining participants are commonly reported [1, 3, 5–9]. If online interventions are to be an effective public health tool, efforts to address these issues are needed so that interventions have sufficient reach to have a real-world impact [10].

The personalisation of study materials (where individuals are referred to by their name) is one technique that has been found to increase response rates [11–14]. Compared to generic invitations to engage in research, the receipt of personalised materials has been shown to increase participation, including in studies conducted in an online environment [11–14]. However, this research has been restricted to survey research, predominately of a cross-sectional nature. For studies that require a far greater deal of commitment on behalf of the participant, such as randomised controlled trials, personalisation of study materials may not result in increased participation rates. In some circumstances, personalisation of recruitment materials for randomised controlled trial may not be possible, due to a lack of personal information available at the time of recruitment. However, in other circumstances, for example in studies targeting individuals with chronic diseases (where national registries containing contact information exist) or in studies employing setting-based recruitment methods (such as workplaces) it may be possible to personalise recruitment materials and doing so may help to boost response rates [11–14].

This study aimed to examine the impact of personalised study invitation emails on the response rates of potentially eligible breast cancer survivors for participation in a randomised controlled trial. The randomised trial itself examined the relative efficacy of three online computer-tailored interventions (differing in delivery schedule only) designed to increase participation in physical activity over a three month period. The trial period was for 6 months, with assessments occurring at baseline, 3 months and 6 months post-baseline. Participation was entirely by distance with all assessments conducted online via the study website.

## Methods

### Design

The current study is a nationally-based quasi-randomised 2-arm controlled trail. Ethical approval was obtained from the Human Research Ethics Committee at Central Queensland University, Australia (H13/07-126). The trial is registered with the Australian and New Zealand Clinical Trials Registry (registration number: ACTRN12613001220752).

### Participants and procedure

The study was conducted between June and August 2014 at Central Queensland University. English proficient female breast cancer survivors who were over 18 years of age, who had finished active cancer treatment, had no contraindications to exercise and were not already participating in 150 min of moderate-vigorous aerobic activity accumulated across at least 5 days a week were eligible to participate. To recruit participants, members (*n* = 43,150) of a breast cancer organisation in Australia, who had agreed to be contacted about breast cancer- related research opportunities, were e-mailed by the organisation about the upcoming randomised trial on behalf of the research team. Volunteers include both male and female member of the general community. The e-mail contained information about the study (study aims, procedures and eligibility criteria) and an instruction to complete a ‘permission to pass on contact details form’ if they thought they were eligible and agreed to be contacted by the research team. No reminder emails were sent.

The breast cancer organisation provided the research team with the contact information of members who had agreed to be contacted in relation to the randomised controlled trial. This information was provided once a week over an eight week period, as not all members responded equally fast to the request. Upon receiving the contact information each week the research team sent one group a personalised email (i.e., addressing them with first and last name) and the other half a generic email (i.e., addressing them as ‘dear member’). The email detailed the study aims, the study eligibility criteria and directed participants to the study website where they could view more information about the study and consent to participate by completing the eligibility questionnaire. The greeting line (personalised or generic) was the only difference between the e-mails sent to each group (see Additional file 1). All participants were sent a reminder email 2–3 weeks after the initial e-mail was sent, which was personalised in the same way as the original e-mail.

### Group allocation

Group allocation was based on last name. Each week the contact information of potential participants was provided to the research team in an excel spreadsheet. This was sorted based on last name and split into two groups (personalised email versus generic email). To reduce any potential bias associated with this, the group receiving the personalised e-mail was switched each week (i.e., in the first week the personalised email was sent to the first half of participants in the excel sheet and in the second week the personalised email was sent to the second half of participants in the excel sheet, and so on for proceeding weeks). All participants were blinded to this process. The project team was not blinded.

### Analysis

Potential participants were classified as ‘responders’ if they completed the eligibility questionnaire. This was determined by cross-referencing contact details (i.e., full name and email address) collected during eligibility screening with information provided by the breast cancer organisation. To explore the association between personalisation and the likelihood of responding a chi-square analysis was performed in Stata using the cs epitab command, which computes both the test statistic and risk ratio.

## Results

**Fig. 1:**
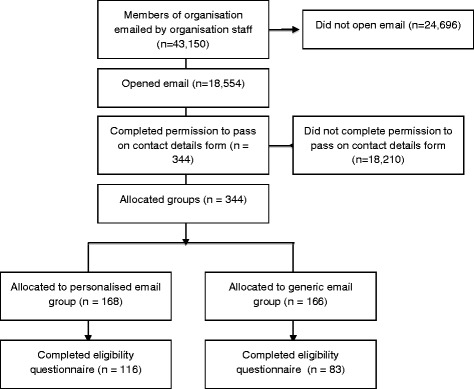
Study flow chart

A diagram illustrating participant flow through the trial is displayed in Fig. 1. Of the 43,150 members emailed on behalf of the research team, 18,554 (43 %) opened the email, and 344 (1.85 % of 18,554) completed the ‘permission to pass on contact details form’. Out of the 344 potential participants contacted by the research team, 199 (58 %) responded to the invitation request, and 181 were deemed eligible and went on to participate in the RCT. A significant association between personalisation and response status was found, with a greater proportion of participants sent the personalised e-mail responding than those sent the generic email (116 (69 %) vs 83 (50 %); ***Χ***^2^ = 12.58, *p* = 0.01). Overall, the likelihood of responding for those that received a personalised email was 1.5 times greater than for those that received a generic email (risk ratio = 1.51, 95 % CI = 1.18–1.93).

## Discussion

Previous research has shown that the personalisation of study invitations significantly increases participant response rates to epidemiology-based research [11–14]. In the current study, a similar effect was observed, even though the level of commitment requested from participants was markedly higher compared to the previous studies. An important difference in response rate was observed (i.e., 1.5 times higher), even though all participants had already received one e-mail about the study from the breast cancer organisation and the sample contained those already interested in breast cancer related research. Taken together, these findings suggest that personalisation may be a useful and potentially powerful tool that can be utilised when recruiting participants into epidemiological and experimental studies in order to boost response rates.

There are some limitations of the study that should be considered when interpreting the results. First, for convenience, the method of allocation used was not truly random and may have introduced unintentional bias. For example, assigning participants based on last name can result in some ethic groups being disproportionately assigned [15]. We did attempt to reduce the potential of this occurring by switching the block of participants that received the personalised email each week, however some bias may still have been introduced. Second, the research team was not blinded to group allocation. While this introduces another potential source of bias [15], it is proposed that this is unlikely an issue in the current study given that all potential participants were unknown to the research team, there was no face-to-face contact and the outcome measure was objective (i.e., whether the eligibility questionnaire was submitted) and not open to interpretation. Of note, participants were blinded to group allocation and not aware of the study aims, which is a strength of the study. Finally, due to privacy restrictions we do not have participant characteristic data for non-responders or those deemed ineligible to participate, and as a result are unable to determine the number of potentially eligible non-responders or explore differences in response rates based on participant characteristics. This data would be useful for examining the generalizability of our findings, especially given that very few (1.8 %) of the potential participants contacted by the breast cancer organisation agreed to be contacted by the research team. While this low participation rate was likely partially due to ineligibility (since organisation list members included both men and women and people with and without a history of cancer) there may have been significant differences between responders and non-responders that were eligible and this would have implications for the generalizability of our findings. Nonetheless, the findings do provide evidence that personalisation was a useful tool for recruiting eligible participants in our study. Of note, no differences in eligibility were found based on whether a personalised invitation was sent and the vast majority of participants that completed the eligibility questionnaire (91 %) were deemed eligible and went on to participate in the RCT. Thus, given that personalisation significantly increased the odds of completing the eligibility questionnaire, it ultimately increased the odds of participating in the trial.

In light of our research findings it is recommended that researchers consider if the personalisation of study materials is both possible and appropriate for enhancing recruitment in their studies. Unfortunately, personalisation is not possible when names from the sampling frame are not available or are unobtainable. Further, personalisation may not be appropriate when the researcher has no known relationship to the potential participants or when the subject matter is of a sensitive nature [12]. In the case of the former, introduction by a known third party is recommended.

## Conclusion

As personalisation of recruitment materials may help to increase response rates this strategy should be considered when recruiting participants into randomised trials.

## Additional file

Additional file 1:
**Non-personalised email template.** (DOCX 15 kb)
