# Erratum to: Do personalised e-mail invitations increase the response rates of breast cancer survivors invited to participate in a web-based behaviour change intervention? A quasi-randomised 2-arm controlled trial

**DOI:** 10.1186/s12874-015-0095-x

**Published:** 2015-11-20

**Authors:** Camille E. Short, Amanda L. Rebar, Corneel Vandelanotte

**Affiliations:** Freemasons Foundation Centre of Men’s Health, School of Medicine, University of Adelaide, Level 7, South Australian Health and Medical Research Institute, North Terrace Adelaide, 5000 Australia; Physical Activity Research Group, School of Human Health and Social Sciences, Central Queensland University, Building 18, Bruce Highway, Rockhampton, 4702 QLD Australia

## Erratum

Since the publication of this article [1], we have been made aware of two errors in the manuscript.

First, it was reported that 344 participants were allocated to one of two groups. This was a typo consistent throughout the article, and should have been 334 (168 intervention, 166 control – which was reported correctly).

Secondly, the RR calculation was based on the transposed table, which produced the correct p value and OR, but incorrect RR (1.38 instead of 1.51). As such, it was reported that: “those sent the personalised email were 1.5 times (95 % CI = 1.18–1.93) more likely to respond than those sent the generic email.” This should have read: “those sent the personalised email were 1.4 times (95 % CI = 1.15–1.66) more likely to respond than those sent the generic email.”

## References

[1] Short CE, Rebar AL, Vandelanotte C (2015). Do personalised e-mail invitations increase the response rates of breast cancer survivors invited to participate in a web-based behaviour change intervention? A quasi-randomised 2-arm controlled trial. BMC Med Res Methodol.

