# Supplementary material for: Do personalised e-mail invitations increase the response rates of breast cancer survivors invited to participate in a web-based behaviour change intervention? A quasi-randomised 2-arm controlled trial
Source: BMC Med Res Methodol. 2015 Aug 19;15:66. doi: 10.1186/s12874-015-0063-5 (PMC4545569; doi:10.1186/s12874-015-0063-5)
Supplement: Additional file 1: — Non-personalised email template. (DOCX 15 kb) [file 12874_2015_63_MOESM1_ESM.docx]

**Non-personalised email template**

Dear member,

Thank you for your interest in the iMove More for Life Study.

The study aims to evaluate whether the provision of physical activity resources online via a specially designed website can help breast and cancer survivors engage in regular physical activity.

Breast cancer survivors not currently undergoing active treatment (radiotherapy and/or chemotherapy) and not currently participating in moderate-vigorous physical activity more than 150 minutes a week are eligible to participate.

If you would like to participate in the study all you need to do is click on the link below. It will take you to the study website where you can view more information about the study and complete the eligibility questionnaire. If you are eligible, we will process your enrolment and provide you with instructions on how to complete the first of four questionnaires and how to access your personalised physical activity advice.

<http://www.mm4l.org.au/>

Thank you very much for your interest,

Kind regards,

Camille Short, PhD

iMove More for life project co-ordinator

**Personalised email template**

Dear <first name> <last name>,

Thank you for your interest in the iMove More for Life Study.

The study aims to evaluate whether the provision of physical activity resources online via a specially designed website can help breast and cancer survivors engage in regular physical activity.

Breast cancer survivors not currently undergoing active treatment (radiotherapy and/or chemotherapy) and not currently participating in moderate-vigorous physical activity more than 150 minutes a week are eligible to participate.

If you would like to participate in the study all you need to do is click on the link below. It will take you to the study website where you can view more information about the study and complete the eligibility questionnaire. If you are eligible, we will process your enrolment and provide you with instructions on how to complete the first of four questionnaires and how to access your personalised physical activity advice.

<http://www.mm4l.org.au/>

Thank you very much for your interest,

Kind regards,

Camille Short, PhD

iMove More for life project co-ordinator
